# Evolution of Cooperation Patterns in Psoriasis Research: Co-Authorship Network Analysis of Papers in Medline (1942–2013)

**DOI:** 10.1371/journal.pone.0144837

**Published:** 2015-12-11

**Authors:** Gregorio González-Alcaide, Jinseo Park, Charles Huamaní, Isabel Belinchón, José M. Ramos

**Affiliations:** 1 Department of History of Science and Documentation, Universitat de València, Valencia, Spain; 2 Korea Institute of Science and Technology Information (KISTI), Deajeon, South Korea; 3 Instituto Nacional de Salud, Lima, Perú; 4 Department of Dermatology, Hospital General Universitario de Alicante, Alicante, Spain; 5 Department of Medicine, Universidad Miguel Hernández de Elche, Alicante, Spain; 6 Department of Internal Medicine, Hospital General Universitario de Alicante, Alicante, Spain; Wake Forest School of Medicine, UNITED STATES

## Abstract

**Background:**

Although researchers have worked in collaboration since the origins of modern science and the publication of the first scientific journals in the eighteenth century, this phenomenon has acquired exceptional importance in the last several decades. Since the mid-twentieth century, new knowledge has been generated from within an ever-growing network of investigators, working cooperatively in research groups across countries and institutions. Cooperation is a crucial determinant of academic success.

**Objective:**

The aim of the present paper is to analyze the evolution of scientific collaboration at the micro level, with regard to the scientific production generated on psoriasis research.

**Methods:**

A bibliographic search in the Medline database containing the MeSH terms “psoriasis” or “psoriatic arthritis” was carried out. The search results were limited to articles, reviews and letters. After identifying the co-authorships of documents on psoriasis indexed in the Medline database (1942–2013), various bibliometric indicators were obtained, including the average number of authors per document and degree of multi-authorship over time. In addition, we performed a network analysis to study the evolution of certain features of the co-authorship network as a whole: average degree, size of the largest component, clustering coefficient, density and average distance. We also analyzed the evolution of the giant component to characterize the changing research patterns in the field, and we calculated social network indicators for the nodes, namely betweenness and closeness.

**Results:**

The main active research clusters in the area were identified, along with their authors of reference. Our analysis of 28,670 documents sheds light on different aspects related to the evolution of scientific collaboration in the field, including the progressive increase in the mean number of co-authors (which stood at 5.17 in the 2004–2013 decade), and the rise in multi-authored papers signed by many different authors (in the same decade, 25.77% of the documents had between 6 and 9 co-authors, and 10.28% had 10 or more). With regard to the network indicators, the average degree gradually increased up to 10.97 in the study period. The percentage of authors pertaining to the largest component also rose to 73.02% of the authors. The clustering coefficient, on the other hand, remained stable throughout the entire 70-year period, with values hovering around 0.9. Finally, the average distance peaked in the decades 1974–1983 (8.29) and 1984–2003 (8.12) then fell over the next two decades, down to 5.25 in 2004–2013. The construction of the co-authorship network (threshold of collaboration ≥ 10 co-authored works) revealed a giant component of 161 researchers, containing 6 highly cohesive sub-components.

**Conclusions:**

Our study reveals the existence of a growing research community in which collaboration is increasingly important. We can highlight an essential feature associated with scientific collaboration: multi-authored papers, with growing numbers of collaborators contributing to them, are becoming more and more common, therefore the formation of research groups of increasing depth (specialization) and breadth (multidisciplinarity) is now a cornerstone of research success.

## Introduction

Psoriasis is a chronic inflammatory disorder, characterized by skin lesions such as erythemas, papules, scaly patches and plaques. The lesions appear in any number of forms, with a pronounced variability in clinical manifestations and degrees of intensity. The five main types of psoriasis are plaque (the most common form), guttate, inverse, pustular, and erythrodermic. Psoriasis has also been associated with several immune disorders, chief among them psoriatic arthritis, but also ulcerative colitis, Crohn's disease, diabetes mellitus and cardiovascular disease, along with an increased risk of certain cancers [1,2]. It can also have a negative impact on health-related quality of life, similar to or greater than other major diseases [3]. Psoriasis prevalence estimations vary from 0.5% to 4.6%, with wide variations depending on age, sex, latitudinal location and ethnicity [4,5]. Although a diverse array of treatments is available, psoriasis is a complex, multi-factorial pathology, and wide gaps remain in our knowledge of its exact etiology [6].

In this context, it is vital to foster research, particularly collaborative projects that integrate different theoretical and methodological approaches, helping to generate the critical mass of knowledge necessary to spur significant advances in our understanding of the disease, together with the translation of that knowledge into clinical practice.

Although researchers have worked in collaboration since the origins of modern science and the publication of the first scientific journals in the eighteenth century, this phenomenon has acquired exceptional importance in the last several decades. Since the mid-twentieth century, new knowledge has been generated from within an ever-growing network of investigators, working cooperatively in research groups across countries and institutions. The research group may be considered the basic organizational unit of science, despite having a frequently informal nature that transcends organizational and institutional structures [7–9]. The field of bibliometrics has developed a number of indicators that help to measure scientific collaboration in a given discipline or area of knowledge. Social network analysis, in turn, can complement bibliometric analysis, identifying the existing research groups and relational structures of the scientific community that support the generation of knowledge.

Different bibliometric studies have examined scientific publications on psoriasis. Pavlovsky et al. [10] analyzed relevant documents collected in the Medline database in 1993–2007, observing that the notable increase in research has led to a “better understanding of psoriasis immunopathology”. Likewise, Schoeffel et al. [11] used the Web of Science database to identify the 10 most productive journals and authors and to analyze international production and collaboration in the area. Ram and Paiwal [12] went further, using Medline to identify the most productive, core journals that published papers on psoriasis in 1960–2009, and then analyzing the distribution of documents among the journals according to Bradford’s law of scattering. More specifically, Jamshidi et al. [13] studied documents on psoriatic arthritis that were indexed in the Web of Science in 1989–2009, incorporating the study of international collaboration and citations received by the documents into their analysis of scientific production.

The objective of the present study is to analyze the collaboration networks and their evolution with regard to psoriasis research at an individual level (co-authorships), and to identify the active research groups in the area. As a basis for our analysis, we used the documents indexed in Medline over a 70-year period, from 1942 to 2013.

## Methods

Our study was carried out in two phases:

Phase 1. Identification of the group of documents under study and standardization of the bibliographic data

Medline was chosen as the study database due to its status as the main reference source in the health sciences, its free access, and its Medical Subject Headings (MeSH) thesaurus, which facilitates a precise identification and analysis of document contents. We first retrieved documents containing the MeSH terms [“psoriasis” or “psoriatic arthritis”]. For our calculation of indicators, we limited the search results to the main document types that present original research results: articles, reviews and letters. No chronological limitation was set, so all documents collected in the database from 1942 to 2013 were analyzed. The search was run on 23 December 2014 using the PubMed platform.

Once the group of documents under study was established, we carried out a standardization process for the document types and author signatures. With regard to the former aspect, Medline generally assigns more than one document type to each paper, so we first isolated the letters and the reviews (which normally also appear as journal articles), and then we unified the different entries corresponding to clinical trials (clinical trial phase I, II, III, IV; controlled clinical trial; and randomized controlled trial). In addition, it was necessary to differentiate all the authors who had the same name, and to standardize the variations in signatures from single authors. We thus carried out a manual review of all signatures, checking institutional affiliations in case of doubt. The main discrepancies we found were caused by one or more first or last names being included, the authors’ first names being either spelled out or abbreviated to the initials, and typos.

Once the authors’ names were standardized, we determined all of the existing co-authorship ties and their frequency. A co-authorship is defined as the joint signature of any document by two specific authors, so the number of co-authorships present in a given paper will depend on the number of authors who sign it. Thus, the same co-authorship may occur *n* times in a large collection of documents, making it possible to establish different thresholds or intensities of collaboration. To process the bibliographic information we used Microsoft Access, and we used Bibexcel software to create files with the co-authorship ties, which we quantified for processing by the analytical programs and network visualization (http://homepage.univie.ac.at/juan.gorraiz/bibexcel/index.html) [14]. All of the data used to carry out the study, including the information downloaded from the database as well as that derived from the treatment of the bibliographic entries, were deposited in the open access public repository The Dataverse Project (https://dataverse.harvard.edu/). The data collected in the current study do not include private or patient records/information because it is a study of existing data and records from an open bibliographic database.

Phase 2. Determination of bibliometric indicators and social network analysis

The following bibliometric indicators were obtained and disaggregated by decade in order to see the evolution of the scientific collaboration:

- Number of authors and signatures (scientific contributions)
- Co-author mean: average number of co-authors per document
- Degree of multi-authorship: proportion of documents with *n* authors (documents with 1 author, 2–5 authors, 6–9 authors, and ≥10 authors)

With specific regard to author-based indicators, we examined the following:

- Productivity of authors, according to different thresholds (authors with 1 document, 2–9 documents, and ≥10 documents)
- Degree of continuous participation and publication in the discipline and incorporation of new researchers, according to the categories introduced by Price and Gürsey [15], which classify authors as *transients* (authors publishing in a given year but neither before nor after), *newcomers* (authors publishing in and after the given year but never before), *terminators* (authors publishing before and in the given year but never after) and *continuants* (authors publishing before, in and after the given year).

With regard to the social network analysis, the following indicators were obtained to characterize the evolution of the overall size of the network and the patterns of scientific collaboration observed therein:

- N vertices: the number of authors making up the network.
- N links: the number of co-authorship links. Both unique and repeating links were identified.
- Average degree: average number of collaborators per author.
- Size of the giant or largest component: the highest number of authors connected directly or indirectly, considering all links and without applying any collaboration threshold, within the entire network. The absolute number of vertices (authors) in the giant component is given, along with the percentage that they represent with regard to the total number of authors in the network. In addition, we have constructed a figure with multiple graphs showing the evolution of the giant component in order to analyze the structure of the network (in the periods 1974–1983, 1984–1993, 1994–2003, and 2004–2013) and contextualize it with the major events in psoriasis research.
- Percentage of isolates: researchers who are not connected with any other researcher.
- Network density: proportion between the number of real links in the network and the maximum number of links that are theoretically possible.
- Clustering coefficient: calculated according to the measure proposed by Watts and Strogatz, as the average of the local clustering coefficients of all the nodes, where the local clustering coefficient of each node is the proportion of real connections between it and its neighbors, compared with the number of all links that could possibly exist between them [16].
- Average distance: average number of intermediaries between nodes, on the shortest path connecting them in the network.

To complement the data derived from the indicators, a co-authorship network was constructed in order to identify the main research clusters that are currently active in the area. For this purpose, only the co-authorships from the most recent decade (2004–2013) were used. Co-authorship frequency was quantified, and a threshold of 10 or more co-authored papers was applied to reduce the number of nodes and links, focusing the analysis on the main collaborative links. In the network, nodes represent authors, and links represent co-authorships; a higher intensity of collaboration is reflected through greater thickness of the links. With regard to this co-authorship network (2004–2013), we have calculated two of the most commonly used measures in network analysis to determine the centrality of the nodes:

- Betweenneess. This indicator assesses the extent to which each node plays the role of intermediary or bridge due to its location along the shortest path between other nodes of the network.
- Closeness. This indicator evaluates the distance between a given node and the rest of the nodes in the network; it is calculated as the sum of the shortest distances from the node being analyzed to all of the other nodes.

To calculate these indicators, we used the Pajek program for visualization and network analysis (http://mrvar.fdv.uni-lj.si/pajek/), using the Kamada-Kawai algorithm for the visual representation of the co-authorship network with the main active research clusters that exist in the area (in 2004–2013) [17]. Two features are reflected in the network. The size of the nodes is proportional to the sum of the two calculated centrality indicators: betweenness and closeness. The degree of continuity is reflected through the use of different colors: yellow nodes present the most consolidated scientific production, having participated in one or more publication in at least nine years of the decade analyzed; green nodes present a high degree of participation, as they are authors with 6–8 years of participation in the decade studied; and red nodes are characterized by a lower level of participation in the publications, having participated in no more than five in the 10-year period.

To show the evolution of the giant component, we used the Gephi program because of its great analytical power to construct and visualize large networks, using the ForceAtlas 2 algorithm [18]. Each color represents a different modularity, which was also calculated by Gephi, that is, the division of the network into communities with dense interconnections within them and sparser connections with other communities (we set the option “randomize”, “use edge weights” and the resolution 1.0). The concept of modularity to detect communities was introduced by Newman and Girvan in 2004 and is one of the most widely used measures to detect communities in weighted networks as co-authorship networks [19,20].

## Results

**Fig 1:**
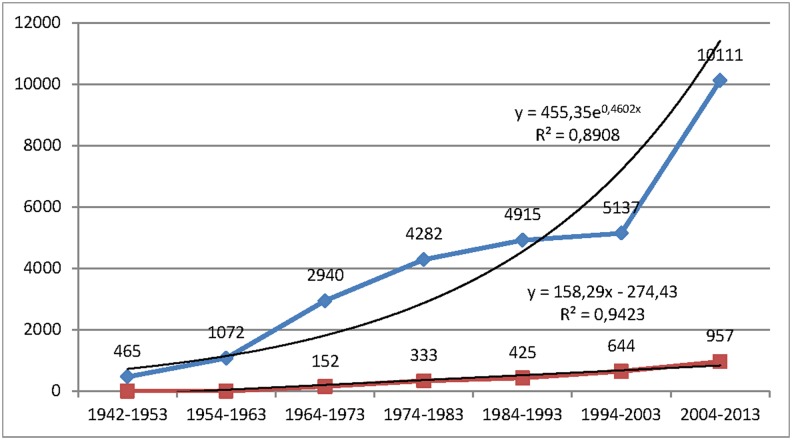
Diachronic evolution of the number of documents. Evolution of the number of documents and clinical trials on psoriasis research indexed in the Medline database.

A total of 28,670 documents were analyzed: 76.34% (n = 21,887) were articles; 13.88% (n = 3981), reviews; and 9.77% (n = 2802), letters. We excluded 776 documents (editorials, news, congresses and interviews) from the analysis. Fig 1 shows the diachronic evolution of the number of documents by decade of publication, which reflects a trend of exponential growth (R^2^ = 0.89). The growth in the last decade in the study period (2004–2013) is especially noteworthy; the number of published documents doubled that of the previous decade. It is also interesting to highlight that 2,511 clinical trials were published, with a trend of linear growth (R^2^ = 0.94) (Fig 1).

The documents were published in 2,460 journals. The three most productive were the *British Journal of Dermatology* (n = 2,037), the *Journal of the American Academy of Dermatology* (n = 1,119) and the *Journal of Investigative Dermatology* (n = 1090). Forty-two journals published 100 or more documents; 302 journals, between 10 and 99; 1,017 journals, between 2 and 9; and 1,096 journals, a single paper.

All in all, 48,899 authors participated, of whom 69.39% (n = 33,930) published a single work; 27.76% (n = 13,573) signed 2–9 papers, and 2.85% (n = 1,396) participated in 10 or more. The percentage of authors that published 10 or more papers has gradually increased, up to 2.46% in the last decade of the study period, while the percentage of authors signing a single paper has stabilized around 70%–73% in the last several decades. With regard to collaboration, the co-author mean increased progressively over the course of the study period, reaching 5.17 authors per document in 2004–2013. Collaboration on articles was greater (5.96 in the same decade) than for letters (4.38) or reviews (3.26) (Table 1).

**Table 1:** Bibliometric indicators of production and collaboration for documents on psoriasis research indexed in the Medline database.

Decade	N docs	N authors	% authors with 1 doc	% authors with >9 docs	N signatures	Co-author mean	N docs (journal articles)	N authors (journal articles)	N signatures (journal articles)	Co-author mean (Journal articles)
1942–1953	459	522	83.14	0.00	647	1.41	459	522	647	1.41
1954–1963	1,070	1,330	79.25	0.30	1,867	1.74	1,064	1,327	1,861	1.75
1964–1973	2,896	3,635	74.25	0.85	5,998	2.07	2,747	3,561	5,753	2.09
1974–1983	4,219	5,972	70.34	1.99	11,189	2.65	3,764	5,643	10,259	2.72
1984–1993	4,877	8,958	71.67	1.58	16,410	3.36	3,873	7,946	13,889	3.59
1994–2003	5,096	11,972	73.93	1.56	21,208	4.16	3,490	10,018	16,777	4.81
2004–2013	10,053	25,168	71.86	2.46	51,961	5.17	6,490	20,483	38,708	5.96
TOTAL	28,670	48,899	69.39	2.85	109,280	3.81	21,887	42,157	87,894	4.01

It is worth noting that papers with many co-authors (multi-authored documents) have increased dramatically. In 1974–1983, documents with 6–9 authors only constituted 5.22% of the total, whereas in 2004–2013, they accounted for 25.77%. Moreover, 10.28% of the documents in the last decade of study had 10 or more authors, compared to about 1% in the previous 20 years (Table 2).

**Table 2:** Distribution of the number of authors in documents on psoriasis research indexed in Medline.

Decade	N docs with 1 author	%	N docs with 2–5 authors	%	N docs with 6–9 authors	%	N docs with ≥10 authors	%
1942–1953	316	68.84	143	31.15	0	0.00	0	0.00
1954–1963	571	53.36	495	46.26	4	0.37	0	0.00
1964–1973	1,171	40.43	1,695	58.53	26	0.90	4	0.14
1974–1983	1,228	29.11	2,763	65.49	220	5.22	8	0.19
1984–1993	901	18.47	3,339	68.46	587	12.04	50	1.02
1994–2003	726	14.25	3,100	60.83	1,059	20.78	211	4.14
2004–2013	955	9.50	5,474	54.45	2,591	25.77	1,033	10.28
TOTAL	5,868	20.47	17,009	59.33	4,487	15.65	1,306	4.55

With regard to the degree of continuity of the authors and the incorporation of new investigators over the period spanning 2005 to 2012, there was a constant rise in the number of continuants, which broke the 1000 mark in 2009. Newcomers have also steadily entered into the network; although their numbers gradually decreased from 2005 to 2012, they have generally outnumbered the terminators, except in the most recent years, where the quantification process favors the presence of terminators more strongly (Table 3).

**Table 3:** Classification of authors with regard to their degree of continuity in publishing documents on psoriasis research indexed in Medline

Year	N Transients	N Newcomers	N Terminators	N Continuants
2005	1,383	863	143	415
2006	1,464	749	176	631
2007	1,509	748	291	833
2008	1,633	617	388	858
2009	1,962	674	400	1,076
2010	1,970	566	587	1,131
2011	2,276	473	736	1,120
2012	2,572	399	1,187	935

Among the network indicators (Table 4), the average degree has continuously increased, up to 10.97 in 2004–2013. The size and the number of authors pertaining to the largest component have grown as well, incorporating 73.02% of all authors in the last decade studied, while the percentage of isolates has diminished to marginal values of about 1%.

**Table 4:** Network indicators for documents on psoriasis research indexed in Medline.

Indicator	1942–1953	1954–1963	1964–1973	1974–1983	1984–1993	1994–2003	2004–2013
N vertices	279	963	3,045	5,476	8,634	11,705	24,858
Average degree	1.53	2.13	2.89	4.13	5.2	7.11	10.97
Size of the largest component. N vertices (%)	10 (3.58%)	16 (1.66%)	140 (4.60%)	1336 (24.40%)	4353 (50.42%)	6870 (58.69%)	18151 (73.02%)
% isolates	46.55%	27.59%	16.23%	8.30%	3.62%	2.23%	1.23%
Density	0.00551817	0.00221717	0.0009494	0.00075421	0.00060206	0.0006074	0.0004414
Watts-Strogatz Clustering Coefficient	0.88496732	0.8906824	0.86428218	0.87325918	0.88338413	0.89705532	0.8886843
N links with value = 1	194	904	3,805	9,483	19,135	36,320	113,615
N links with value > 1	20	123	595	1,823	3,303	5,288	22,751
Average distance	1.29	1.45	4.3	8.29	8.12	6.18	5.25

**Fig 2:**
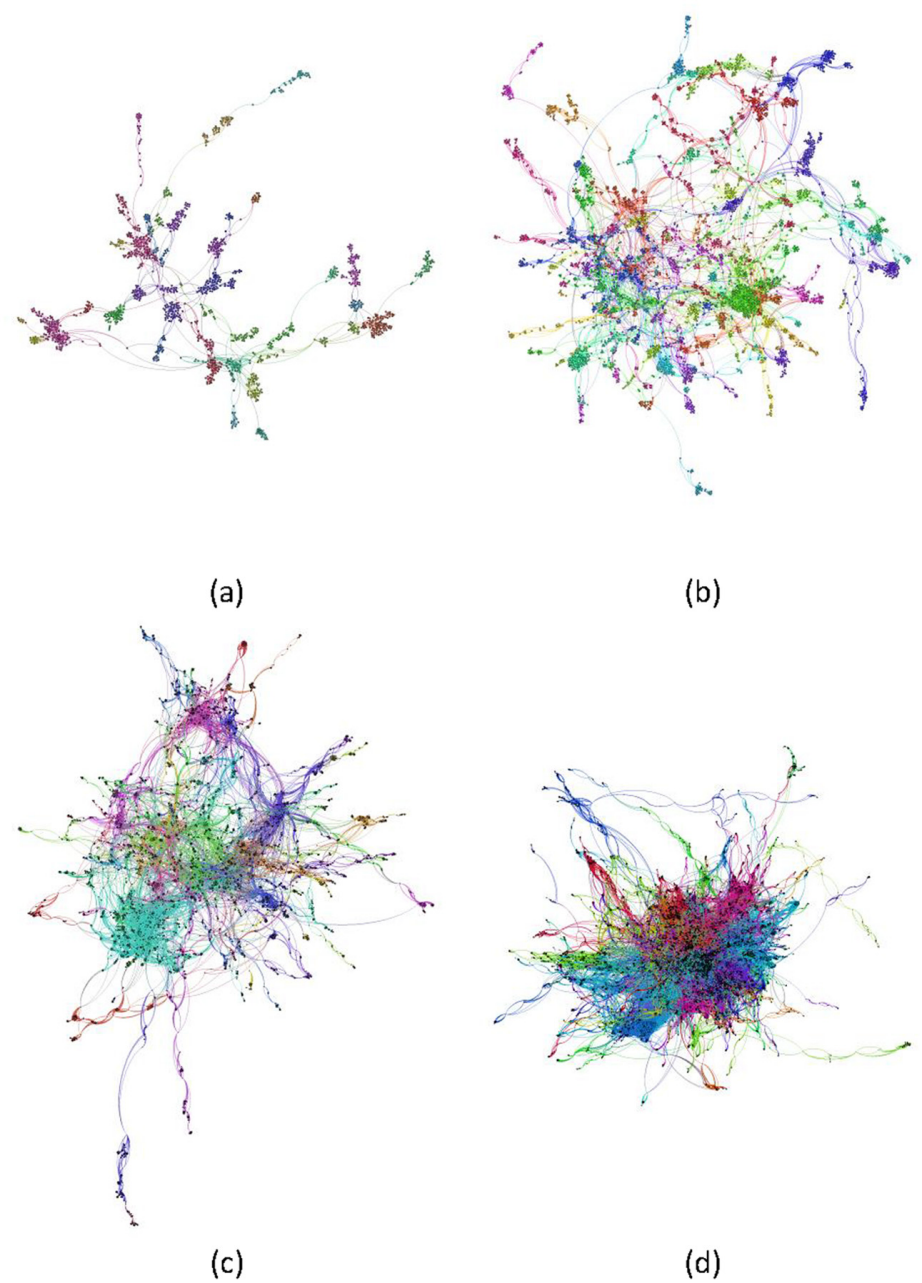
Evolution of giant component in co-authorship network. Giant component by decades in the co-authorship network for documents on psoriasis research indexed in Medline.

The execution of the algorithm of modularity on the nodes making up the giant component allowed us to identify 35 communities in the 1973–1983 period, 63 in 1984–1993, 60 in 1994–2003, and 98 in 2004–2013. The visual representation of the evolution of the giant component by decade (Fig 2) shows that until 1994–2003, it is possible to clearly make out different communities, some of them quite dense—if not highly interconnected, while in the most recent decade we observe the greatest degree of interconnection between the communities in the center of the network, with only a few communities that are clearly differentiated in the network periphery.

Network density gradually decreased over the study period, although it did remain stable in the decades 1984–1993 and 1994–2003, before dropping most notably in 2004–2013. The clustering coefficient was stable throughout the study period, with values hovering around 0.9. Finally, the average distance reached its highest values in 1974–1983 (8.29) and 1984–1993 (8.12), before a sensible decrease in the two decades that follow, when it finally dropped to 5.25 in 2004–2013.

**Fig 3:**
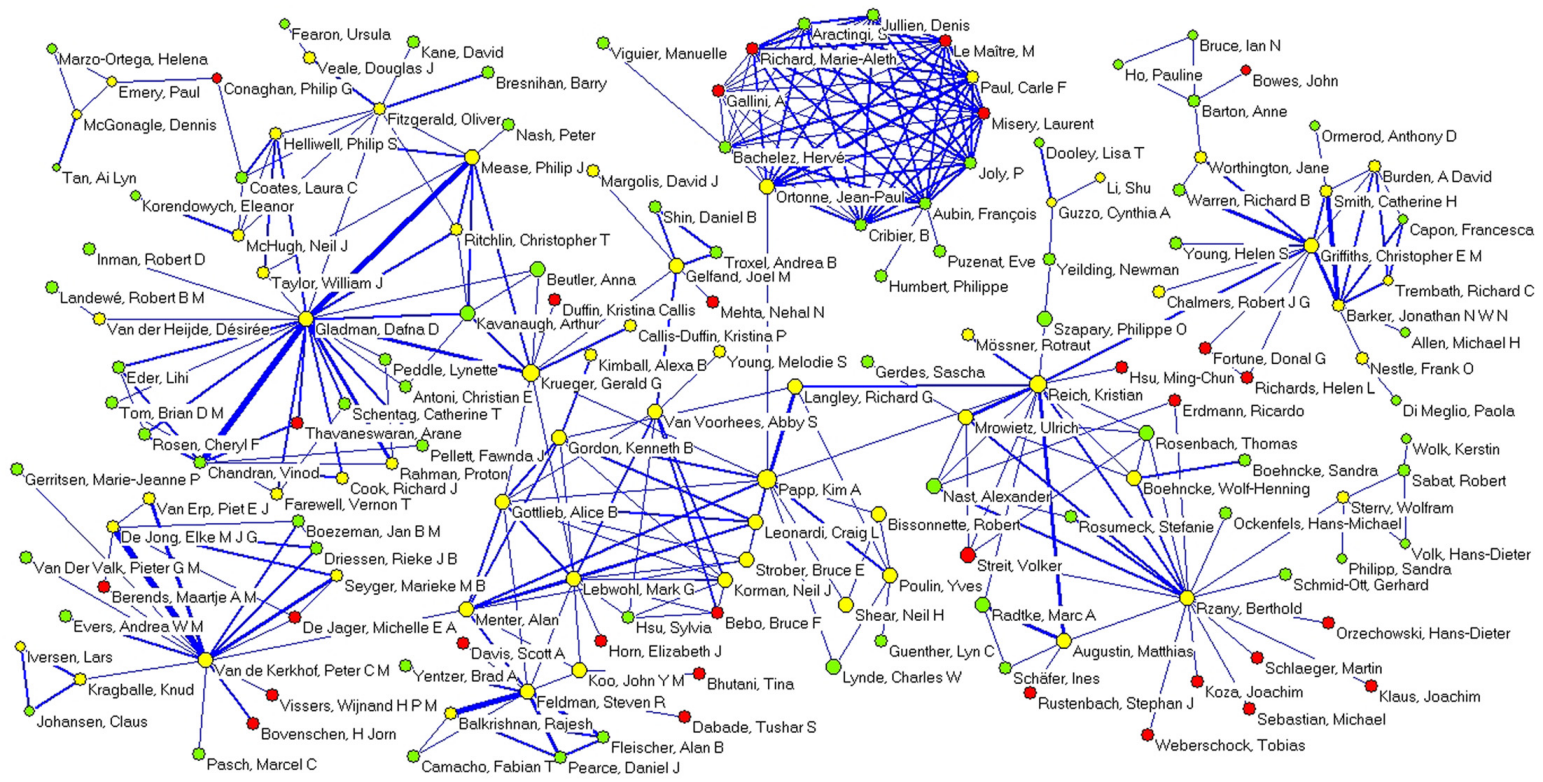
Co-authorship network giant component (collaboration threshold ≥ 10 co-authored papers). Giant component in the co-authorship network for documents on psoriasis research indexed in Medline (2004–2013)

**Fig 4:**
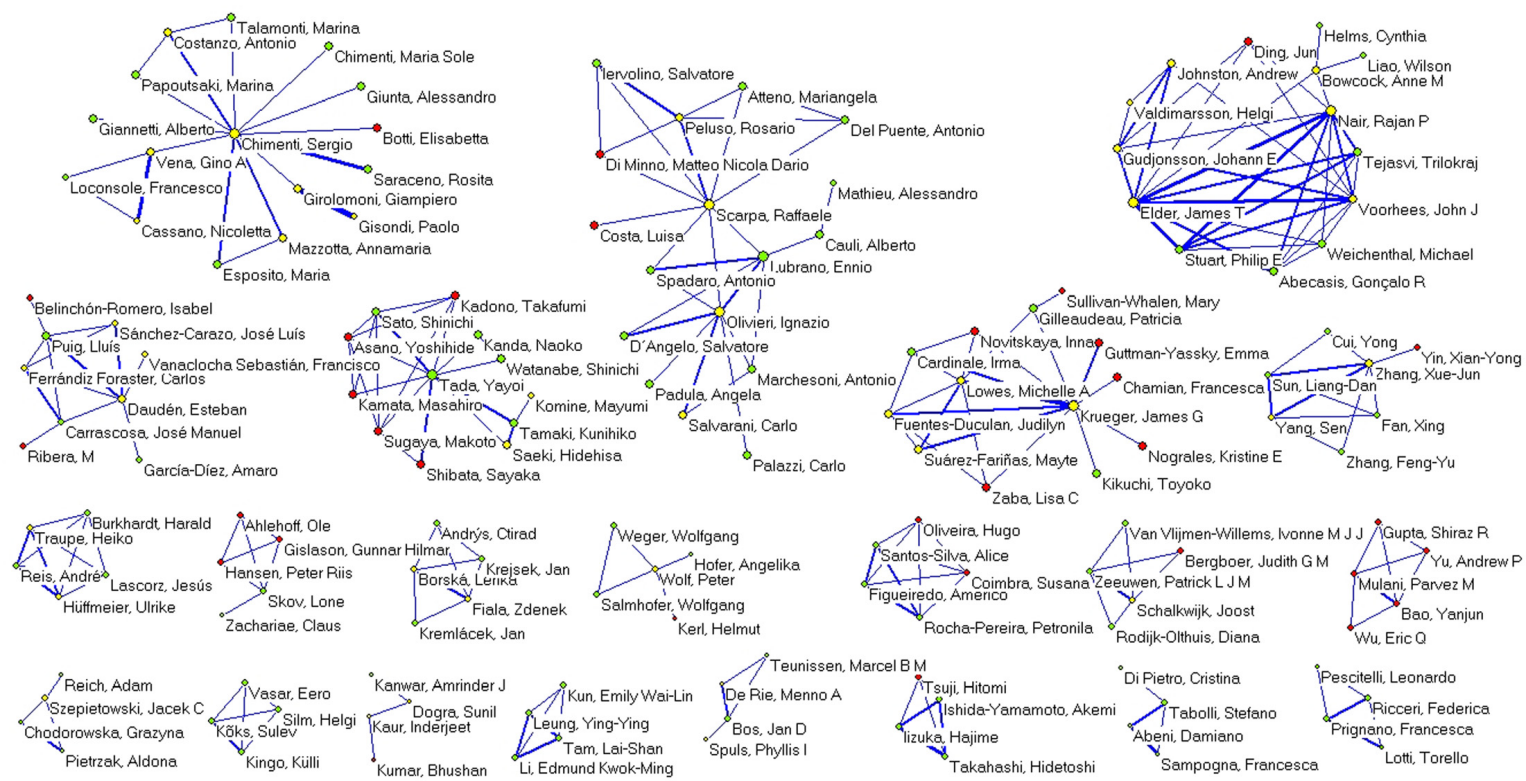
Co-authorship network, secondary clusters (collaboration threshold ≥ 10 co-authored papers). Secondary clusters in the co-authorship network for documents on psoriasis research indexed in Medline (2004–2013)

The construction of the co-authorship network (collaboration threshold ≥ 10 co-authored papers) revealed a giant component made up of 161 researchers. Six very cohesive sub-components are also apparent (Fig 3), as are 22 other prominent clusters composed of between 4 and 17 researchers (Fig 4). Nine other smaller clusters have 3 researchers each, while 23 clusters have just 2 members.

Table 5 presents the 10 most prominent authors with regard to the measures of centrality betweenness and closeness, in order to evaluate the overall relevance of the different nodes (network authors). Kim A. Papp, Kristian Reich, and Gerald G. Krueger occupy the top three positions for both indicators, while most of the other authors ranked in the top 10 have prominent positions in their respective subcomponents of the constructed co-authorship network (Fig 3).

**Table 5:** Measures of centrality for the nodes of the co-authorship network on psoriasis research indexed in Medline (2004–2013).

Rank	Author	Betweenness	Author	Closeness
1	Papp, Kim A.	0.009112	Papp, Kim A.	0.042128
2	Reich, Kristian	0.007856	Reich, Kristian	0.038077
3	Krueger, Gerald G.	0.006193	Krueger, Gerald G.	0.038004
4	Gladman, Dafna D.	0.003789	Gottlieb, Alice B.	0.037288
5	Menter, Alan	0.003487	Menter, Alan	0.035421
6	Griffiths, Christopher E. M.	0.003314	Langley, Richard G.	0.035045
7	Van de Kerkhof, Peter C. M.	0.002850	Gordon, Kenneth B.	0.033904
8	Rzany, Berthold	0.002537	Leonardi, Craig L.	0.033789
9	Ortonne, Jean-Paul	0.002421	Strober, Bruce E.	0.033674
10	Fitzgerald, Oliver	0.001443	Ortonne, Jean-Paul	0.032945

## Discussion

### Synthesis of the findings

#### Bibliometric approach: increase in multi-authored scientific production

Researchers tend to organize in collaborative patterns. Understanding the essential features of cooperative practices through bibliometric indicators that analyze the scientific production of a discipline or area of knowledge can lead to a better comprehension of the social environment in which research develops. Our study has confirmed the clear trend, also observed by previous research, of increasing scientific production on psoriasis, particularly after the year 2000. Pavlovsky et al. [10] speculate that this phenomenon could be rooted in the development of new biological treatments for psoriasis that do not displace traditional treatments, or perhaps in the existence of a high level of scientific evidence. This latter possibility is supported by Price’s theory on exponential growth in scientific literature; applying those principles to psoriasis research reveals that the field is in a mature stage and has contributed important advances in knowledge and treatment for the disease [21]. Indeed, one of the main paths for knowledge generation is through clinical trials, and this document type constituted no less than 10.6% of the analyzed documents published in 1994–2003. This figure stands in contrast to the 0.75% of clinical trial papers published on retinoblastoma [22] and the 2% published on leishmaniasis [23]. In the case of psoriasis, not only has the number of documents increased (at a rate of 97.3% between the decades 1994–2003 and 2004–2013), but also the number of authors (110.2% increase) and authorships (145%).

The co-author mean is an indicator of the general degree of collaboration in the area, and it has not stopped growing over the last several decades, a fact that can be explained by the benefits of collaboration, the mobility of researchers, and the spectacular development of communications systems [7,24]. On the other hand, the literature has also warned that author lists may be inflated due to the phenomenon of unjustified hyper-authorship, which stems in part from pressure to publish and which, according to some studies, now represents a significant proportion of authorships in the biomedical area [25,26]. With regard to the effects of collaboration at a co-author level, some references have associated the increase in co-authorships with a higher degree of acceptance among scientific journals as well as an increase in productivity or citation rates, especially when it comes to international collaborations [25, 27–31].

The degree of multi-authorship is a useful indicator in analyzing the evolution of the quantity of authors involved in the development of the studies and in the dissemination of the results, shedding light on the research effort mobilized for the performance of studies and the drafting of papers. In our study, the indicator reveals a noticeable increase in the number of papers signed by many different authors, another inherent feature of scientific collaboration. This fact responds to the rise in interdisciplinary research, which utilizes the knowledge of authors from a variety of different areas to explore complex problems from a multidisciplinary perspective [32]. In clinical research, multicenter studies and clinical trials may also contribute to the increase in multi-authorship; this type of research is increasingly common due to the level of evidence that it provides and the potential to translate the results to practice [33,34]. Other factors can also be mentioned, such as institutional policies that aim to foster collaboration as a way to raise the profile of their researchers. This practice responds to evidence showing that multi-authored manuscripts are more likely to be accepted in journals with high impact factors or in the core journals of the field, raising the potential for high citation rates [35]. The formation of multidisciplinary and multicenter groups—increasingly broad and at the same time specialized—is therefore one of the key elements that ensures the progress of knowledge and the translation of research results.

Our study suggests that there is great human potential in the field of psoriasis research, in the form of a stable community of researchers that lends continuity to the topic. First of all, there are an important number of *big producers* (authors with over 9 papers)—2.85% of the authors, which is well above the values seen in other disciplines that are comparable in terms of scientific production. For example, big producers only account for 1.45% of authors in the area of alcohol research [36]. A high number of continuants (authors who published before and after the years under study) was also observed. These aspects are essential in the establishment of collaborative ties and the formation of consolidated research clusters, both of which favor the efficient development of research and the integration of new members [37,38].

#### Social Network Analysis: an approach to structural and cooperative research relationships

Social network analysis opens the door to new perspectives for the study and characterization of scientific collaboration, highlighting the network’s global features and identifying the skeleton of the network—the most active research components—by analyzing the interactions and the role played by the main authors within them. The identification of reference researchers in a field through network analysis can be useful in determining the most successful cooperative practices and in identifying potential collaborators. With regard to the average degree, this measure facilitates an analysis into the number of different co-workers of the researchers into the research community, determining if collaboration is more or less extensive, while the distribution of the links (considering different thresholds of co-authorship) provides insight into the extent to which authors have established stable links with other researchers or have participated in more sporadic collaborations. Both of these parameters mark the evolution of the breadth (number of collaborators) and the intensity of the collaborations (frequency of co-authorships with a single researcher).

With regard to the breadth of collaboration, the fact that the average number of co-authors has doubled in the past 20 years is striking, even if (as observed in previous studies), this collaboration is concentrated among sporadic partnerships. Indeed, stable and consolidated collaborators are still few in number, usually limited to just one co-author or a limited group of linked co-authors [39,40]. This suggests that the increase in sporadic collaborators may respond to factors such as a larger presence of occasional authors who contribute to the specialty without fully dedicating their work to it, or the participation of authors that provide concrete technical or instrumental support, for example, experts in specific methodologies, lab technicians or statisticians. The existence of a high number of transient authors is common in all scientific disciplines, although if this number rises too high, it could be an obstacle to the consolidation of the discipline or reflect the absence of a strong intellectual base of researchers dedicated to the field [41]. Gutiérrez-Vela et al. [42] interpreted this to be the case in the area of regenerative periodontal surgery, where 79.6% of all authors had only participated on one document. In telepathology, a recent and relatively unconsolidated field, 76.2% of authors are also transient [43]. In any case, both of these values are significantly higher than the 69.3% of transient authors observed in the present study.

The maximum frequency of co-authorships that we observed is far above those observed in other, less consolidated areas, such as health management [44], and approximate the values seen in the study of diseases such as leishmaniasis or in areas such as oncology [40,45]. The maintenance of strong ties (i.e., repeated co-authorships) has been associated with better scientific performance than a series of weak ties with many different researchers [46,47], which constitutes an additional facet from bibliometric analyses that attests to the importance of big producers for a scientific discipline.

The percentage of authors that belong to the largest component reflects the degree of integration of the researchers within the whole of the scientific community under study. In this sense, the value that we observed in the last decade of study (73%) is very similar to that calculated for the research network on HIV and HPV (70%) [48], although somewhat lower that observed in the networks focusing on leishmaniasis (79.7%), Chagas (84.1%), and coronary heart disease (95%) [39,40,49], which suggests the existence of a wide consensus with regard to the topics covered, but at the same time a few specific topic areas within the field of research that are covered by independent groups without ties to other researchers. Conversely, the percentage of isolate authors corresponds to the authors who do not work collaboratively; this value has gradually fallen to the marginal values seen today.

With regard to the evolution of the giant component and the modularity, two aspects are particularly significant: first, the considerable increase in the number of communities identified in the decades 1984–1993 and 2004–2013, which almost double those observed in the preceding periods. Second, the stability of the 1994–2003 period compared to the previous decade is noteworthy, as the number of communities identified barely increases, despite the considerable increase in authors making up the giant component; this period seems to be characterized by the reinforcement of existing communities rather than by the appearance of new communities. The communities identified can be interpreted as homogeneous research clusters that share a common interest in a given study topic or line of research. It is worth noting that in the study carried out and in relation to the communities identified, the evolution in their number does not evolve in a similar way to the increase in authors that make up the network or in the collaborative links that connect them, suggesting the importance that the prominent milestones and key discoveries have in driving the generation of new knowledge in a discipline, as well as the interest in modularity as a measure to characterize this aspect. The increase in the communities identified in the 1984–1993 decade coincides with the first milestone in the treatment of psoriasis, the advent of phototherapy (with or without psoralen) and immunomodulatory drugs such as cyclosporine or methotrexate, among others [50–52]. From 2000 on, it is worth noting the introduction of anti-tumor necrosis factor (anti-TNF) alpha therapies, which block the biologic activity of psoriasis. The first clinical trial was published in 2001, testing the efficacy of infliximab, and from the year 2003, a number of other published clinical trials focused on other anti-TNF agents, including etanercept or adalimumab. More recently, other biological drugs, such as ustekinumab, ixekizumab, tofacitinib, secukinumab and briakinumab, have targeted immunological mediators of the disease [53].

The average distance is a measure that can be associated with how fast information and innovations circulate in the network. The high values of this indicator observed in the decades 1974–1983 (8.3) and 1984–1993 (8.1) stand out, and can be interpreted as correlating to a nascent stage in the development of the network, with distant research clusters linked by few interconnections. Over the next two decades, the average distance decreases significantly, to values similar to those seen in other scientific co-authorship networks [54]. Thus, it appears that the psoriasis network has matured and multiplied its collaborative ties, which facilitates a faster and more effective dissemination of information, methods, and innovations within the field’s research community [55].

The clustering coefficient quantifies the degree of connection maintained between adjacent nodes for each node in the network, determining the extent to which they tend to bunch together to form cohesive groups, with a high density of ties among them. The high clustering coefficients observed, together with the average degree values observed, establish the psoriasis network as a *small world* network, a configuration that scientific co-authorship networks assume [16,46,56]. One of the most notable practical implications of this kind of network is the existence of a few key nodes, which make it possible to get to any other node in the network with a minimum number of intermediaries, despite the fact that most nodes are not directly connected. These key nodes are considered to be some of the most important scientific agents, favoring network cohesion and the advancement of knowledge. Another defining feature of small world networks is the likelihood that two nodes that are connected indirectly through an intermediary will eventually establish a direct tie. This aspect is predictive of the network’s evolution: an author *A* may collaborate separately with authors *B* and *C*, and it is probable that the latter two will end up establishing a collaborative link.

The authors represented in the co-authorship network (2004–2013) were identified by applying a high collaboration threshold to identify the stable and consolidated collaborative relationships. The resulting clusters can therefore be considered the skeleton that articulates all research in the area [49] and supports the rest of the network, which may be much larger, including transient authors, newcomers and researchers with less intense collaborative ties that do not meet the threshold imposed for the construction of the network. According to diverse studies, consolidated research groups perform better and achieve the highest degrees of citation, so it is important to establish the conditions that favor the creation of these groups and clusters [57].

Most of the sub-components of the largest component, along with many other research clusters identified, present centralized, star-shaped topologies, with a single investigator occupying a prominent position that brings together other authors. This figure suggests that the most common way that research groups are organized depends on the existence of a principal author, who acts as its leader. According to network theory research, these authors are characterized by their collaborative ties with a large number of researchers [58], their status as highly productive authors [59], and the lowest geodesic distances registered in the network, which confers on them a leadership role due to their capacity to interact more directly and rapidly with other investigators [55]. Thus, these are researchers of reference, who are responsible for articulating the development of research in the area and facilitating wider collaborative relationships, the cohesion of scientific community, and the integration of new authors in the research groups. This collective reflects the hierarchical structure of research development; indeed, one study on co-authorships in the area of chemical engineering found that many clusters represented the collaborating authors of a full professor, usually the department chairman [60].

In other cases, the research clusters appear as highly cohesive structures with a high degree of connectivity among the authors, none of which stands out from the rest. This is the case of the cluster at the top of Fig 3, which is among the clusters with the highest number of average co-authors per paper and which also stands out for the large number of papers signed by many collaborators. This probably reflects an entirely different model for organizing researchers in the production of knowledge, less stratified and more democratic and distributive. Alternatively, the pattern may be attributable to factors such as research specialization, with the participation of numerous investigators, or a markedly multidisciplinary approach, as noted by previous studies [61]. In any case, some papers have warned that structures that are excessively closed off or homogenous, with few external links to other clusters, could constitute an obstacle to the integration of new researchers and innovations, and hence to the advancement of knowledge [62,63].

It is also worth noting that with regard to the constructed networks, some research clusters have peripheral or lateral ties, which probably represent different stages of development within the group. Newer authors may be assuming leadership positions alongside more consolidated researchers, or new research nuclei could be emerging. Future lines of research should deepen the analysis of the topologies and structures in the co-authorship networks, through qualitative methodologies or network analyses based on block-modeling, in order to determine to what extent they respond to different cooperative patterns or behaviors [64–66] and which organizational forms are most effective in generating new knowledge [67].

With regard to the measures of centrality of the nodes, the highest ranked researchers in betweenness and closeness are those who occupy central positions in the sub-components they belong to, which confirms the interest in the combined use of both indicators to identify the most prominent authors, who lead their research cluster and play an important role in the network. Some authors also stand out for their role as a bridge, connecting different research clusters; these investigators are incredibly important to their field because they facilitate the dissemination of information and new ideas, the application of research methodologies and the exchange of resources [61,68].

### Conclusions and possible implications of our study results

The main findings of this study are as follows:

1. The bibliometric indicators showed a high degree of maturity in psoriasis research, with considerable scientific production and good transfer of knowledge due to the existence of a growing research community. Scientific collaboration is increasingly important, as evidenced by both the steady rise in the average number of authors per paper and the quantity of multi-authored works.
2. Scientific collaboration is a polifacetic phenomenon. Network-based indicators open new perspectives in the study of collaboration, emphasizing interactions and the role played by individual researchers within the whole scientific discipline under analysis. In this sense, the rise in the number of collaborators among investigators of psoriasis is evident, along with a progressive integration and interconnection that are characteristic of small world scientific networks, which stand out for their high level of interconnection and cohesion. The main research clusters have also been identified, as have the authors of reference who present a greater intensity of co-authorships.
3. We can highlight an essential feature associated with scientific collaboration: multi-authored papers, with growing numbers of collaborators contributing to them, are becoming more and more common, therefore the formation of research groups of increasing depth (specialization) and breadth (multidisciplinarity) is now a cornerstone of research success.

### Limitations and future research

The present study must assume the general limitations inherent to the study of scientific collaboration through co-authorships. These have already been described in previous studies, which point out the importance of informal research collaborations that may not be reflected in the list of co-authors and the possible distorting effect of honorific authorships, among other aspects [69–71]. It is also important to keep in mind that scientific collaboration can be measured at different levels (for example, institutional, national, cross-disciplinary or cross-sectoral), and that there are other forms of collaboration whose final result is not a scientific publication, such as the co-direction of doctoral theses, the development of reports or research projects, or the joint organization of activities such as scientific congresses, among others [72,73].

The present study offers a broad panorama of the evolution of scientific collaboration in psoriasis research, based on the study of co-authorship networks. Scientific collaboration is a complex and changeable reality, around which there are some certainties but also a vast and unexplored territory. Given the fact that research groups constitute the basic organizational structure around which the scientific community is organized, future lines of work should focus on the processes for identifying them and the bibliometric analysis of their scientific production, determining patterns of publication, collaboration and impact among the groups’ members. The analysis of communities through the concept of modularity, the knowledge generated within them, and their relation to the evolution of the number of authors and co-authorship links should be a specific focus of analysis in future studies [19,20]. Likewise, other variables can be integrated into network analyses on coauthorships, such as the incidence of multicenter, multidisciplinary, or multicountry collaborations, among others, studying whether these variables favor the cohesion of the networks or if the agents that belong to them, which take part in this type of collaboration, occupy more prominent positions (for example with regard to the measures of centrality) or favor other features, like the connectivity between different research clusters.

## References

[1] Griffiths CE, Barker JN. Pathogenesis and clinical features of psoriasis. Lancet. 2007;370(9583):263–71. 10.1016/S0140-6736(07)61128-3 17658397

[2] Richard MA, Barnetche T, Horreau C, Brenaut E, Pouplard C, Aractingi S, et al Psoriasis, cardiovascular events, cancer risk and alcohol use: evidence-based recommendations based on systematic review and expert opinion. J Eur Acad Dermatol Venereol. 2013;27(Supplement 3):2–11. 10.1111/jdv.12162 23845148

[3] Rapp SR, Feldman SR, Exum ML, Fleischer AB Jr, Reboussin DM. Psoriasis causes as much disability as other major medical diseases. J Am Acad Dermatol. 1999;41:401–7. 10459113 10.1016/s0190-9622(99)70112-x

[4] Goff KL, Karimkhani C, Boyers LN, Weinstock MA, Lott JP, Hay RJ, et al The Global Burden of Psoriatic Skin Disease. Br J Dermatol. 2015;172(6):1665–8. 10.1111/bjd.13715 25645671

[5] Lebwohl M. Psoriasis. Lancet. 2003;361:1197–204. 12686053 10.1016/S0140-6736(03)12954-6

[6] Chandra A, Ray A, Senapati S, Chatterjee R. Genetic and epigenetic basis of psoriasis pathogenesis. Mol Immunol. 2015;64(2):313–23. 10.1016/j.molimm.2014.12.014 25594889

[7] Glänzel W. Coauthorship patterns and trends in the sciences (1980–1998): a bibliometric study with implications for database Indexing and search strategies. Library Trends. 2002;50(3):461–73.

[8] Whitfield J. Collaboration: Group theory. Nature. 2008;455(7214):720–3. 10.1038/455720a 18843335

[9] Wutchy S, Jones BF, Uzzi B. The increasing dominance of teams in production of knowledge. Science. 2007;316(5827):1036–9. 10.1126/science.1136099 17431139

[10] Pavlovsky L, Mimouni FB, Hodak E, David M, Mimouni D. From basic research to biological treatments: psoriasis publications over the past 15 years. Clin Exp Dermatol. 2009;34(5):e91–3. 10.1111/j.1365-2230.2008.03199.x 19438559

[11] Schoeffel N, Mache S, Al-Mutawakel K, Quarcoo D, Scutaru C, Groneberg DA, et al A new view on psoriasis research efforts. J Eur Acad Dermatol Venereol. 2010;24(1):85–8. 10.1111/j.1468-3083.2009.03293.x 19453771

[12] Ram S, Paliwal N. Assessment of Bradford Law’s of scattering to psoriasis literature through bibliometric snapshot. DESIDOC J Lib Inf Technol. 2014;34(1):46–56.

[13] Jamshidi AR, Gharibdoost F, Nadji A, Nikou M, Habibi G, Mardani A, et al Presentation of psoriatic arthritis in the literature: a twenty-year bibliometric evaluation. Rheumatol Int. 2013;33(2):361–7. 10.1007/s00296-012-2428-y 22451035

[14] Persson O, Danell R, Wiborg Schneider J. How to use Bibexcel for various types of bibliometric analysis In: Åström F, Danell R, Larsen B, Schneider J, editors. Celebrating scholarly communication studies: A Festschrift for Olle Persson at his 60th Birthday. Leuven, Belgium: International Society for Scientometrics and Informetrics; 2009 pp. 9–24.

[15] Price DS, Gürsey S. Studies in Scientometrics I. Transience and Continuance in Scientific Authorship. Ci. Inf. Rio de Janeiro. 1975;4(1):27–40.

[16] Watts DJ, Strogatz SH. Collective dynamics of “small-world” networks. Nature. 1998;393(6684):409–10. 10.1038/30918 9623998

[17] Kamada T, Kawai S. An Algorithm for Drawing General Undirected Graphs. Information Processing Letters. 1988;31:7–15. 10.1016/0020-0190(89)90102-6

[18] Jacomy M, Venturini T, Heymann S, Bastian M (2014) ForceAtlas2, a Continuous Graph Layout Algorithm for Handy Network Visualization Designed for the Gephi Software. PLoS ONE 9(6): e98679 10.1371/journal.pone.0098679 24914678 PMC4051631

[19] Malliaros FD, Vazirgiannis M. Clustering and community detection in directed networks: a survey. Physics Reports. 2013;533(4):95–142. 10.1016/j.physrep.2013.08.002

[20] Newman ME. Girvan M. Finding and evaluating community structure in networks. Physical Review E. 2004;69:026113 10.1103/PhysRevE.69.026113 14995526

[21] Price DJS. Little Science, Big Science. New York: Columbia University Press; 1963.

[22] Zhao ZG, Guo XG, Xu CT, Pan BR, Xu LX. Bibliometric analysis on retinoblastoma literatures in PubMed during 1929 to 2010. Int J Ophthalmol. 2011;4(2):115–20. 10.3980/j.issn.2222-3959.2011.02.01 22553624 PMC3340704

[23] Ramos JM, González Alcaide G, Bolaños Pizarro M. Bibliometric analysis of leishmaniasis research in Medline (1945–2010). Parasit Vectors. 2013;6:55 10.1186/1756-3305-6-55 23497410 PMC3602049

[24] Bozeman B, Fay D, Slade CP. Research collaboration in universities and academic entrepreneurship: the-state-of-the-art. J Technol Transf. 2013;38:1–67. 10.1007/s10961-012-9281-8

[25] Flanagin A, Carey LA, Fontanarosa PB, Phillips SG, Pace BP, Lundberg GD, et al Prevalence of articles with honorary authors and ghost authors in peer-reviewed medical journals. JAMA. 1998;280(3):222–4. 10.1001/jama.280.3.222 9676661

[26] Kennedy MS, Barnsteiner J, Daly J. Honorary and ghost authorship in nursing publications. J Nurs Scholarsh. 2014;46(6):416–22. 10.1111/jnu.12093 24930670

[27] Braun T, Glänzel W, Schubert A. Publication and cooperation patterns of the authors of neuroscience journals. Scientometrics. 2001;51(3):499–510. 10.1023/A:1012774206340

[28] Gordon MD. A critical reassessment of inferred relations between multiple authorship, scientific collaboration, the production of papers and their acceptance for publication. Scientometrics. 1980;2(3):193–201. 10.1007/BF02016697

[29] Katz JS, Hicks D. How much is a collaboration worth? A calibrated bibliometric model. Scientometrics. 1997;40(3):541–54. 10.1007/BF02459299

[30] Narin F, Stevens K, Whitlow ES. Scientific cooperation in Europe and the citation of multinationally authored papers. Scientometrics. 1991;21(3):313–23. 10.1007/BF02093973

[31] Persson O, Glänzel W, Danell R. Inflationary bibliometric values: the role of scientific collaboration and the need for relative indicators in evaluative studies. Scientometrics. 2004; 60(3):421–32. 10.1023/B:SCIE.0000034384.35498.7d

[32] Nabout JC, Parreira MR, Teresa FB, Carneiro FM, da Cunha HF, Ondei LS, et al Publish (in a group) or perish (alone): the trend from single- to multi-authorship in biological papers. Scientometrics. 2015;102:357–64. 10.1007/s11192-014-1385-5

[33] Chow DS, Ha R, Filippi CG. Increased rates of authorship in radiology publications: a bibliometric analysis of 142,576 articles published worldwide by radiologists between 1991 and 2012. AJR Am J Roentgenol. 2015;204(1):W52–77. 10.2214/AJR.14.12852 25539275

[34] Tsay M, Yang Y. Bibliometric analysis of the literature of randomized controlled trials. J Med Libr Assoc. 2005;93(4):450–8. 16239941 PMC1250321

[35] Sahu SR, Panda KC. Does the multi-authorship trend influence the quality of an article? Scientometrics. 2014;98:2161–8. 10.1007/s11192-013-1127-0

[36] González Alcaide G, Castelló Cogollos L, Castellano Gómez M, Agulló Calatayud V, Aleixandre Benavent R, Álvarez FJ, et al Scientific publications and research groups on alcohol consumption and related problems worldwide: authorship analysis of papers indexed in Pubmed and Scopus databases (2005 to 2009). Alcohol Clin Exp Res. 2013;37(suppl. 1):381–93. 10.1111/j.1530-0277.2012.01934.x 22974198

[37] Ioannidis JPA, Boyack KW, Klavans R. Estimates of the continuously publishing core in the scientific workforce. Plos ONE. 2014;9(7):e101698 10.1371/journal.pone.0101698 25007173 PMC4090124

[38] Wu B, Zhao F, Yang S, Suo L, Tian H. Characterizing the evolution of collaboration network 2nd ACM workshop on Social Web Search and Mining. Hong Kong: SWSM’09; 2009 p. 33–40.

[39] González Alcaide G, Park J, Huamaní C, Gascón J, Ramos Rincón JM. Scientific authorships and collaboration network analysis on Chagas disease: papers indexed in PubMed (1940–2009). Rev Inst Med Trop Sao Paulo. 2012;54(4):219–28. 22850995 10.1590/s0036-46652012000400007

[40] González-Alcaide G, Huamaní C, Park J, Ramos JM. Evolution of coauthorship networks: worldwide scientific production on leishmaniasis. Rev Soc Bras Med Trop. 2013;46(6):719–27. 10.1590/0037-8682-0207-2013 24474013

[41] Schubert A, Glänzel W. Publication dynamics: models and indicators. Scientometrics. 1991;20(1):317–31. 10.1007/BF02018161

[42] Gutiérrez-Vela MM, Díaz-Haro A, Berbel-Salvador S, Lucero-Sánchez A, Robinson-García N, Cutando-Soriano A. Bibliometric analysis of research on regenerative periodontal surgery during the last 30 years. J Clin Exp Dent. 2012;4(2):e112–8. 10.4317/jced.50646 24558535 PMC3908794

[43] Della Mea V. 25 years of telepathology research: a bibliometric analysis. Diagn Pathol. 2011;6(Suppl 1):S26 10.1186/1746-1596-6-S1-S26 21489197 PMC3073220

[44] Zhang C, Yu Q, Fan Q, Duan Z. Research collaboration in health management research communities. BMC Med Inform Decis Mak. 2013;13:52 10.1186/1472-6947-13-52 23617236 PMC3640984

[45] Qi Y, Shao H, Duan Z. Research groups of oncology coauthorship network in China. Scientometrics. 2011;89(2):553–67. 10.1007/s11192-011-0465-z

[46] Abbasi A, Altmann J. On the correlation between research performance and Social Network Analysis measures applied to research collaboration networks. 44th Hawaii International Conference on System Sciences, Koloa, Kauai, Hawai, USA. 2011.

[47] de Montjoye YA, Stopczynski A, Shmueli E, Pentland A, Lehmann S. The Strength of the strongest ties in collaborative problem solving. Sci Rep. 2014;4:5277 10.1038/srep05277 24946798 PMC4064431

[48] Vanni T, Mesa-Frias M, Sanchez-Garcia R, Roesler R, Schwartsmann G, Goldani MZ, et al International scientific collaboration in HIV and HPV: a network analysis. Plos ONE. 2014;9(3):e93376 10.1371/journal.pone.0093376 24682041 PMC3969316

[49] Yu Q, Shao H, He P, Duan Z. World scientific collaboration in coronary heart disease research. Int J Cardiol. 2013;167(3):631–9. 10.1016/j.ijcard.2012.09.134 23068572

[50] Lawrence CM, Marks J, Parker S, Shuster S. A comparison of PUVA-etretinate and PUVA-placebo for palmoplantar pustular psoriasis. Br J Dermatol. 1984;110(2):221–6. 10.1111/j.1365-2133.1984.tb07471.x 6696838

[51] Parker S, Coburn P, Lawrence C, Marks J, Shuster S. A randomized double-blind comparison of PUVA-etretinate and PUVA-placebo in the treatment of chronic plaque psoriasis. Br J Dermatol. 1984;110(2):215–20. 10.1111/j.1365-2133.1984.tb07470.x 6365147

[52] Willkens RF, Williams HJ, Ward JR, Egger MJ, Reading JC, Clements PJ, et al Randomized, double-blind, placebo controlled trial of low-dose pulse methotrexate in psoriatic arthritis. Arthritis Rheum. 1984;27(4):376–81. 10.1002/art.1780270403 6712754

[53] Chaudhari U, Romano P, Mulcahy LD, Dooley LT, Baker DG, Gottlieb AB. Efficacy and safety of infliximab monotherapy for plaque-type psoriasis: a randomised trial. Lancet. 2001;357(9271):1842–7. 10.1016/S0140-6736(00)04954-0 11410193

[54] Newman ME. Coauthorship networks and patterns of scientific collaboration. Proc Natl Acad Sci USA. 2004;101(Suppl 1):5200–5. 10.1073/pnas.0307545100 14745042 PMC387296

[55] Kretschmer H. Author productivity and geodesic distance in bibliographic co-authorship networks, and visibility on the Web. Scientometrics. 2004;60(3):409–20. 10.1023/B:SCIE.0000034383.86665.22

[56] Zare-Farashbandi F, Geraei E, Siamaki S. Study of co-authorship network of papers in the Journal of Research in Medical Sciences using social network analysis. J Res Med Sci. 2014;19(1):41–6. 24672564 PMC3963322

[57] Lambiotte R, Panzarasa P. Communities, knowledge creation, and information diffusion. J Informetr. 2009;3(3):180–90. 10.1016/j.joi.2009.03.007

[58] González Teruel A, González Alcaide G, Barrios M, Abad-García MF. Mapping recent information behavior research: an analysis of coauthorship and co-citation networks. Scientometrics. 2015;103(2):687–705. 10.1007/s11192-015-1548-z

[59] Bales ME, Johnson SB, Keeling JW, Carley KM, Kunkel F, Merrill JA. Evolution of co-authorship in public health systems and services research. Am J Prev Med. 2011;41(1):112–7. 10.1016/j.amepre.2011.03.018 21665073 PMC3677523

[60] Peters HPF, Van Raan AFJ. Structuring scientific activities by co-author analysis. Scientometrics. 1991;20(1):235–55. 10.1007/BF02018157

[61] Bian J, Xie M, Topaloglu U, Hudson T, Eswaran H, Hogan W. Social network analysis of biomedical research collaboration networks in a CTSA institution. J Biomed Inform. 2014;52:130–140. 10.1016/j.jbi.2014.01.015 24560679 PMC4136998

[62] Burt RS. Structural holes: the social structure of competition. Cambridge, MA: University Press; 1992.

[63] Long JC, Cunningham FC, Braithwaite J. Network structure and the role of key players in a translational cancer research network: a study protocol. BMJ Open. 2012;2:e001434 10.1136/bmjopen-2012-001434 22734122 PMC3383981

[64] Bayer AE, Smart JC. Career Publication Patterns and Collaborative “Styles” in American Academic Science. J High Educ. 1991;62(6):613–36. 10.2307/1982193

[65] Crane D. Social structure in a group of scientists: a test of the “invisible college” hypothesis. American Sociological Review. 1969;34(3):335–52.

[66] Velden T, Haque A, Lagoze C. A new approach to analyzing patterns of collaboration in co-authorship networks: mesoscopic analysis and interpretation. Scientometrics. 2010;85(1):219–42. 10.1007/s11192-010-0224-6

[67] Kshitij A, Ghosh J, Gupta BM. Embedded information structures and functions of co-authorship networks: evidence from cancer research collaboration in India. Scientometrics. 2015;102(1):285–306. 10.1007/s11192-014-1310-y

[68] Huang S, Lv T, Zhang X, Yang Y, Zheng W, Wen C. Identifying Node Role in Social Network Based on Multiple Indicators. PLoS ONE. 2014;9(8):e103733 10.1371/journal.pone.0103733 25089823 PMC4121239

[69] Glänzel W, Schubert A. Analyzing scientific network through co-authorship In: Moed HF, Glänzel W, Schmoch U, editors. Handbook of quantitative science and technology research. Dordrecht: Springer; 2005 p. 257–76.

[70] Katz JS, Martin BR. What is research collaboration? Research Policy. 1997;26(1):1–18. 10.1016/S0048-7333(96)00917-1

[71] Laudel G. Collaboration and reward: What do we measure by co-authorships? Research Evaluation. 2002;11(1):3–15. 10.3152/147154402781776961

[72] González Alcaide G, Gómez Ferri J. La colaboración científica: principales líneas de investigación y retos de futuro. Rev Esp Doc Cient. 2014;37(4):e62 10.3989/redc.2014.4.1186

[73] Mali F, Kronegger L, Doreian P, Ferligoj A. Dynamic scientific co-authorship networks In: Scharnhorst A, Börner K, van den Besselaar P, editors. Models of science dynamics, understanding complex systems. Heidelberg: Springer; 2012 p. 195–232.

